# Supplementary material for: Analysis of PPARγ Signaling Activity in Psoriasis
Source: Int J Mol Sci. 2021 Aug 10;22(16):8603. doi: 10.3390/ijms22168603 (PMC8395241; doi:10.3390/ijms22168603)
Supplement: Supplementary file 1 [file ijms-22-08603-s001.zip › Supplemental materials_Analysis of PPARg signaling activity in psoriasis/Pathway models/Models images and html files/Differentiation of psoriatic T cells/49999.html]

prostaglandin E2


# Small Molecule prostaglandin E2

|  |  |
| --- | --- |
| URN | urn:agi-cas:363-24-6 |
| Total Entities | 7 |
| Connectivity | 9002 |
| Name | prostaglandin E2 |
| Description | prostaglandine E2 |
| Class | Endogenous compound |
| Molecular Weight | 352.465080 |
| XLogP | 2.800000 |

---

|  |  |
| --- | --- |
| ChildConcepts | PGD3 |
|  | PGD2 |
|  | PGF2alpha |
|  | PGA2 |
|  | 8-epi-PGF2alpha |
|  | 8-iso-PGE2 |
|  | PGD1 |

---

|  |  |
| --- | --- |
| Pathway | Bradykinin Effects in Inflammation |
|  | Platelet Activation via GPCR Signaling |
|  | Hypothalamic-Pituitary-Adrenal Axis in Acute Phase Response |
|  | Prostaglandin E2 Receptor Signaling in Neurons |
|  | Eicosanoids in Myeloid Cell Activation |
|  | Eicosanoids in Inflammation |
|  | Prostaglandin E2 and Histamine in Gastric Acid Secretion |
|  | Mast-Cells De Novo Synthesized Mediators via IgE Independent Signaling |
|  | Vasodilation Activation |
|  | Vascular Endothelial Cell Activation by Cytokines |
|  | Vascular Endothelial Cell Activation by Blood Coagulation Factors |
|  | Vascular Endothelial Cell Activation by Growth Factors |
|  | Lipolysis Regulation in Adipocyte |
|  | Nociception Expression Targets Signaling |
|  | Vascular Motility |
|  | Ca2+ Flux Regulation |
|  | PTGER1/4 -> Vascular Motility |
|  | PTGER2/3 -> Vascular Motility |
|  | PTGER2/3 -> Inflammation-Related Expression Targets |
|  | IL6 Expression Targets -> Nociception |
|  | IL1B Expression Targets -> Nociception |
|  | NMDA Receptors -> Ca2+/CREB Activation/PGE2 Synthesis |
|  | Luteinizing Hormone in Oocyte Meiotic Resumption |
|  | Prostaglandins Role in Sleep Regulation |
|  | Hematopoietic Cell Lineage: B-cell |
|  | Arterial Hypertension |
|  | T-Cells Differentiation Block in Psoriasis |
|  | Metastatic Colorectal Cancer |
|  | Goblet-Cell Related Mucus Secretion in Asthma |
|  | Osteoclast Activation in Gout |
|  | Monocytes Function in Gout |
|  | IL1B Induced Arthralgia |
|  | Vascular Endothelial Cell Vasodilation in Brain |
|  | Dopamine Action in CSD Progression |
|  | Postsynaptic Neuron Activation |
|  | eNOS (NOS3) Activation via Prostaglandin E2 and Histamine |
|  | Prostaglandin E2 Synthesis Activation via Glutamate in Familial Hemiplegic Migraine |
|  | Neuroinflammation in Amyotrophic Lateral Sclerosis |
|  | Local Estrogen Production in Endometriosis |
|  | Angiogenesis in Endometriosis |
|  | BMP Signaling Impairment in Granulosa Cell in POF |
|  | HPV E5 Regulates EDNRA and EGFR Signaling Pathway in Keratinocytes |
|  | Sertoli Cells Dysfunction Causes Male Infertility |
|  | Aryl Hydrocarbon Receptor/Arachidonic Acid Metabolites Signaling |
|  | Valdecoxib Induced Ischemic Disease |
|  | EGFR Transactivation in Cancer and Non-Cancer Cells |
|  | IL23A and IL17A Provoke Cancer-Associated Inflammation |
|  | HIF1A in Vasculogenic Mimicry of Cancer |
|  | VEGFA Dependent Angiogenesis in Cancer |
|  | Tumor Infiltrating Macrophages in Cancer Progression and Immune Escape |
|  | Myeloid Derived Suppressor Cells in Cancer Immune Escape |
|  | Arachidonic Acid Metabolism |
|  | IL1B -> PGE2 Expression Targets |
|  | ROS and RNS in Vasoconstriction and Vasodilation Regulation |
|  | PPAR Psoriasis |
|  | 1\_Differentiation of psoriatic T cells |
|  | 1\_TNF and IL1B provoke ECM degradation in osteoarthritis |
|  | 1\_Local estrogen production stimulates ectopic endometrium proliferation |
|  | 3\_Enhanced angiogenesis in endometriosis |
|  | 4\_Endothelial cells repress vasoconstriction in hypertension |
|  | 3\_1\_Mucus accumulation in asthma |
|  | 4\_Mucin hyperproduction in goblet and mucous cells in COPD |
|  | 1\_1\_Glutamate overload enforced prostaglandins and NO synthesis in neurons |
|  | 1\_2\_Glutamate overload and NO-mediated vasodilation |
|  | Differentiation of psoriatic T cells |

---

|  |  |
| --- | --- |
| MedScan ID | 1215412 |

---

|  |  |
| --- | --- |
| Alias | Prostaglandins E2 |
|  | propess |
|  | L-PGE2 |
|  | [1-14C]prostaglandin E2 |
|  | (e,z)-(1r,2r, 3r)-7-[3-hydroxy-2-[(3s)-(3-hydroxy-1-octenyl)]-5-oxocyclopentyl]- 5-heptenoic acid |
|  | Prostaglandin E2 |
|  | urinary prostaglandin E2 |
|  | prostin E2 vaginal cream |
|  | [12C]prostaglandin E2 |
|  | (e,z)-(1r,2r, 3r)-7-(3-hydroxy-2-((3s)-(3-hydroxy-1-octenyl))-5-oxocyclopentyl)- 5-heptenoic acid |
|  | prostaglandin E II |
|  | U 12062 |
|  | cerviprime |
|  | prostine |
|  | prostaglandin (PG) E2 |
|  | Prepidil Gel |
|  | (e,z)-(1r,2r,3r,5s)-7-(3-hydroxy-2-((3s)-(3-hydroxy-1-octenyl)-5-oxocyclopentyl)-5-heptenoic acid |
|  | prostin E2 tab |
|  | 363-24-6 |
|  | Prostin |
|  | postaglandin E2 |
|  | (5Z,11alpha,13E,15S)-11,15-Dihydroxy-9-oxoprosta-5,13-dienoate |
|  | PG007 |
|  | dinoprostone gel |
|  | Prostaglandin PGE2 |
|  | l-7-(3-Hydroxy-2-(3-hydroxy-1-octenyl)-5-oxocyclopentyl)-5-heptenoate |
|  | NSC 165560 |
|  | minprostin e 2 |
|  | U-12062 |
|  | (e,z)-(1r,2r,3r,5s)-7-[3-hydroxy-2-[(3s)-(3-hydroxy-1-octenyl]-5-oxocyclopentyl]-5-heptenoic acid |
|  | (15S)-Prostaglandin E2 |
|  | (5z,11a,13e,15s)-11,15-dihydroxy-9-oxoprosta-5,13-dien-1-oic acid |
|  | uPGE2 |
|  | Prepidil |
|  | Dinoprostone |
|  | Dinoprostona |
|  | (e,z)-(1r,2r,3r,5s)-7-(3-hydroxy-2-((3s)-(3-hydroxy-1-octenyl)-5-oxocyclopentyl)-5-heptenoate |
|  | 7-[3-hydroxy-2-(3-hydroxy-1-octenyl)-5-oxocyclopentyl]-5-heptenoic acid |
|  | prostaglandin (PG) E2alpha |
|  | (e,z)-(1r,2r,3r,5s)-7-[3-hydroxy-2-[(3s)-(3-hydroxy-1-octenyl]-5-oxocyclopentyl]-5-heptenoate |
|  | Dinoproston |
|  | (5Z,11-alpha,13E,15S)-11,15-Dihydroxy-9-oxoprosta-5,13-dien-1-oate |
|  | dinoprostone sodium |
|  | L-Prostaglandin E2 |
|  | (5Z,11-alpha,13E,15S)-11,15-Dihydroxy-9-oxoprosta-5,13-dien-1-oic acid |
|  | Cervidil |
|  | uPGE(2) |
|  | primiprost |
|  | prostin R |
|  | 7-[3-hydroxy-2-(3-hydroxy-1-octenyl)-5-oxocyclopentyl]-5-heptenoate |
|  | 11,15 dihydroxy 9 oxoprosta 5,13 dien 1 oic acid |
|  | prostin 3 |
|  | (5Z,11alpha,13E,15S)-11,15-Dihydroxy-9-oxoprosta-5,13-dien-1-oic |
|  | U,12, 062 |
|  | prostarmon E |
|  | PGE2 |
|  | ORG2634 |
|  | dinoprostin |
|  | (e,z)-(1r,2r, 3r)-7-(3-hydroxy-2-((3s)-(3-hydroxy-1-octenyl))-5-oxocyclopentyl)- 5-heptenoate |
|  | l-7-(3-Hydroxy-2-(3-hydroxy-1-octenyl)-5-oxocyclopentyl)-5-heptenoic acid |
|  | prostin E2 vaginal gel |
|  | (5Z,11alpha,13E,15S)-11,15-Dihydroxy-9-oxoprosta-5,13-dienoic acid |
|  | (5z,13e)-(15s)-11alpha,15-dihydroxy-9-oxoprosta-5,13-dienoate |
|  | (5z,13e)-(15s)-11alpha,15-dihydroxy-9-oxoprost-13-enoate |
|  | minprostin |
|  | PGE2alpha |
|  | (e,z)-(1r,2r, 3r)-7-[3-hydroxy-2-[(3s)-(3-hydroxy-1-octenyl)]-5-oxocyclopentyl]- 5-heptenoate |
|  | Dinoprostonum |
|  | Prostenon |
|  | EINECS 206-656-6 |
|  | (5z,11a,13e,15s)-11,15-dihydroxy-9-oxoprosta-5,13-dien-1-oate |
|  | Prostaglandin E2 alpha |
|  | Prostin E2 |
|  | PGE(2) |
|  | prandin E2 |
|  | prostaglandine E2 alpha |
|  | L-Prostaglandine E2 |
|  | prostaglandine E2 |
|  | prostaglandine (PG) E2alpha |
|  | prostaglandine (PG) E2 |
|  | prostaglandin gel |
|  | prostaglandin pessary |
|  | minprostin E2 |
|  | Minprositin E2 |
|  | minproistin E2 |
|  | 5-heptenoic acid, 7-(3-hydroxy-2-(3-hydroxy-1-octenyl)-5-oxocyclopentyl) |
|  | 5-heptenoic acid,7-(3-hydroxy-2-(3-hydroxy-1-octenyl)-5-oxocyclopentyl)-, l |

---

|  |  |
| --- | --- |
| CAS ID | 363-24-6 |

---

|  |  |
| --- | --- |
| Reaxys ID | 2224720 |
|  | 2224721 |
|  | 2224722 |
|  | 2224723 |
|  | 2224724 |
|  | 2421225 |
|  | 2705862 |
|  | 2705863 |
|  | 2819704 |
|  | 2819705 |
|  | 2819706 |
|  | 2819707 |
|  | 2819708 |
|  | 2819709 |
|  | 2819710 |
|  | 2819711 |
|  | 2819712 |
|  | 2819713 |
|  | 2950614 |
|  | 2950615 |
|  | 2950616 |
|  | 2950617 |
|  | 2950618 |
|  | 2950619 |
|  | 2950620 |
|  | 2950621 |
|  | 2950622 |
|  | 2950623 |
|  | 3037904 |
|  | 3037905 |
|  | 3037906 |
|  | 4152491 |
|  | 4152492 |
|  | 4152493 |
|  | 4152494 |
|  | 4152495 |
|  | 4709355 |

---

|  |  |
| --- | --- |
| ChEBI ID | 15551 |

---

|  |  |
| --- | --- |
| PharmaPendium ID | Dinoprostone |

---

|  |  |
| --- | --- |
| HMDB ID | HMDB01220 |
|  | HMDB12580 |
|  | HMDB12601 |
|  | HMDB12638 |
|  | HMDB12777 |
|  | HMDB12855 |

---

|  |  |
| --- | --- |
| KEGG ID | C00584 |

---

|  |  |
| --- | --- |
| InChIKey | XEYBRNLFEZDVAW-ARSRFYASSA-N |

---

|  |  |
| --- | --- |
| Molecular Formula | C20H32O5 |

---

|  |  |
| --- | --- |
| PubChem SID | 134973974 |
|  | 152904 |

---

|  |  |
| --- | --- |
| PubChem CID | 5280360 |

---

|  |  |
| --- | --- |
| IUPAC Name | (Z)-7-[(1R,2R,3R)-3-hydroxy-2-[(E,3S)-3-hydroxyoct-1-enyl]-5-keto-cyclopentyl]hept-5-enoic acid |

---

|  |  |
| --- | --- |
| Rotatable Bond Count | 12 |

---

|  |  |
| --- | --- |
| PharmaPendium Name | Dinoprostone |

---
